# Effects of a high-intensity interval training program versus a moderate-intensity continuous training program on maximal oxygen uptake and blood pressure in healthy adults: study protocol for a randomized controlled trial

**DOI:** 10.1186/s13063-016-1522-y

**Published:** 2016-08-18

**Authors:** Víctor Hugo Arboleda Serna, Elkin Fernando Arango Vélez, Rubén Darío Gómez Arias, Yuri Feito

**Affiliations:** 1 Grupo de Investigación en Actividad Física para la Salud (AFIS), Instituto de Educación Física, Universidad de Antioquia UdeA, Calle 70 No. 52-21, Medellín, Colombia; 2 Facultad Nacional de Salud Pública, Universidad de Antioquia UdeA, Calle 70 No. 52-21, Medellín, Colombia; 3 Department of Exercise Science & Sport Management, Kennesaw State University, 520 Parliament Garden Way, NW, MD 4104 Kennesaw, GA USA

**Keywords:** Blood pressure, Exercise, High-intensity interval training, Randomized controlled trial, VO_2max_

## Abstract

**Background:**

Participation in aerobic exercise generates increased cardiorespiratory fitness, which results in a protective factor for cardiovascular disease and all-cause mortality. High-intensity interval training might cause higher increases in cardiorespiratory fitness in comparison with moderate-intensity continuous training; nevertheless, current evidence is not conclusive. To our knowledge, this is the first study to test the effect of high-intensity interval training with total load duration of 7.5 min per session.

**Methods:**

A randomized controlled trial will be performed on two groups of healthy, sedentary male volunteers (*n* = 44). The study protocol will include 24 exercise sessions, three times a week, including aerobic training on a treadmill and strength training exercises. The intervention group will perform 15 bouts of 30 s, each at an intensity between 90 % and 95 % of maximal heart rate. The control group will complete 40 min of continuous exercise, ranging between 65 % and 75 % of maximal heart rate. The primary outcome measure to be evaluated will be maximal oxygen uptake (VO_2max_), and systolic and diastolic blood pressure will be evaluated as secondary outcome measures. Waist circumference, body mass index, and body composition will also be evaluated.

**Discussion:**

Epidemiological evidence shows the link between VO_2max_ and its association with chronic conditions that trigger CVD. Therefore, finding ways to improve VO_2max_ and reduce blood pressure it is of vital importance to public health.

**Trial registration:**

NCT02288403. Registered on 4 November 2014.

## Background

Maximal oxygen consumption (VO_2max_) provides a measure of the maximal volume of oxygen that the body consumes via the respiratory system and is transported through the bloodstream to be used to release energy in the cell. VO_2max_ is currently deemed the best indicator to assess cardiorespiratory fitness [1, 2] and is considered one of the most important predictors of cardiovascular disease in both men and women, even when taking into consideration smoking history, hypertension, and dyslipidemias. In addition, VO_2max_ is directly related to cardiovascular health, and its improvement has been linked to decreases in risk of death from cardiovascular disease [3]. For every metabolic equivalent gained in VO_2max_, mortality rates have been shown to decrease by about 8–17 % [3–5] (one metabolic equivalent = 3.5 ml/(kg min)). Laukkanen *et al.* [6] studied the association between low levels of VO_2max_ (<27.6 ml/(kg min)) and premature death and found that unfit men had more than twice the risk of overall death (relative risk, 2.76; 95 % confidence interval, 1.43–5.33) and over three times the risk of cardiovascular-disease-related death (relative risk = 3.09; 95 % confidence interval, 1.10–9.56) in comparison with fit men (VO_2max_ >37.1 ml/kg/min).

Moderate-intensity continuous training methods and high-intensity interval training are typically prescribed to increase VO_2max_. The latter has been generally used with athletes; however, this modality has recently been used on untrained individuals with cardiometabolic disorders and cardiovascular disease [7]. Considering the inverse relationship between intensity and duration of exercise, it is important to consider the total volume of the exercise session when prescribing this type of program. High-intensity interval training protocols are typically classified utilizing short intervals (around 30 s) and intensities near maximum (80–100 % peak heart rate), whereas moderate-intensity continuous training programs typically remain between 60 % and 75 % of peak heart rate). In addition, an alternative term has been suggested [7] to denote training programs that utilize intensities over 100 % peak heart rate (sprint interval training). In this study, we will employ a high-intensity interval training protocol of low volume.

The worldwide survey on fitness trends has ranked high-intensity interval training at the top of the top 20 trends for the past two consecutive years [8, 9], which supports use in the general populations, and reveals interest in this training methodology. However, although the benefits of these two modalities are a matter of discussion, comparisons between the two training modalities suggest that high-intensity interval training can produce faster and more significant adaptations in VO_2max_ [10–16]. The results of several meta-analyses have shown slightly higher improvements in VO_2max_ with high-intensity interval training than moderate-intensity continuous training; however, many of these studies used short interventions with few weekly training sessions [17, 18]. In addition, it is important to point out that in a recent meta-analysis on the effects of interval training on VO_2max_, only 4 of the 21 studies that met the inclusion criteria were randomized controlled trials [19]. Milanović *et al.* [20] pointed out the large improvements in VO_2max_ when comparing high-intensity interval training with control subjects who do not exercise (5.5 ml/(kg min)); meanwhile, when comparing high-intensity interval training with aerobic training, this improvement was reduced (1.2 ml/(kg min)).

High blood pressure is one of the most common health disorders; it is associated with an increase in the incidence of all-cause mortality and cardiovascular disease. It has been reported that people with systolic blood pressure between 130 and 139 mmHg, and diastolic blood pressure between 85 and 89 mmHg have a greater risk of cardiovascular events than those who present with optimal blood pressure values (systolic blood pressure <120 mmHg, diastolic blood pressure <80 mmHg), with hazard ratio = 2.5; 95 % confidence interval = 1.6–4.1 for women, and hazard ratio = 1.6; 95 % confidence interval = 1.1–2.2 for men. Likewise, individuals with high blood pressure (systolic blood pressure >140 mmHg, diastolic blood pressure >90 mmHg, or those who use antihypertensive medication) have greater risk of cardiovascular events than individuals with optimal values [21]. Moreover, researchers have found an inverse association between cardiorespiratory fitness and the risk of high blood pressure, where for every 1 ml/(kg min) increase in VO_2max_, the prevalence of hypertension is reduced by 1.04 times (95 % confidence interval, 1.05–1.02) [22]. In their recent meta-analysis looking at the effects of exercise training in blood pressure, Cornelissen and Smart [23] reported decreases in systolic blood pressure and diastolic blood pressure of 3.5 mmHg (95 % confidence interval, 2.3–4.6), and 2.5 mmHg (95 % confidence interval, 1.7–3.2), respectively, with aerobic exercise. Studies comparing continuous training, high-intensity interval training and control subjects who did not exercise and were hypertensive reported significant mean decreases in systolic blood pressure of 8 mmHg in all groups [12], and mean decreases in systolic blood pressure and diastolic blood pressure with high-intensity interval training of 12 and 8 mmHg, respectively, in comparison with continuous training subjects, who obtained non-significant decreases of 4.5 and 3.5 mmHg [24].

Even though exercising is a fundamental aspect in primary prevention, treatment, and control of hypertension, the optimal frequency, intensity, time, and mode of exercise to reduce systolic blood pressure and diastolic blood pressure values are still unclear [25]. In this sense, it is necessary to conduct randomized controlled trials that allow us to examine and confirm the cause–effect relationship between high-intensity interval training and VO_2max_, as well as between high-intensity interval training and blood pressure. Although evidence exists that suggests the benefits of high-intensity interval training in VO_2max_ and blood pressure in individuals with cardiovascular disease [7], the effect of these programs among relatively healthy young men with low levels of physical activity is unclear [7, 19, 20]. Current evidence regarding this topic is limited; thus, this project aims to provide clarity regarding the appropriate dose (frequency, volume, and intensity) to generate significant improvements in VO_2max_ and decreases in systolic and diastolic blood pressure in healthy individuals, as the potential benefits of this type of training are still unclear in this group [26, 27] Taking into account that lack of time is typically the most common cited barrier to engagement in exercise training [28], we believe that this study is novel in its approach, as, to our knowledge, we are the first to examine the effects of a low-volume high-intensity interval training protocol of only 7.5 min per session. Although other studies have utilized less work time per session than this study, those investigations utilized near maximal protocols (≥100 % VO_2max_) and were not randomized control trials [29, 30]. In addition, our comparison group will use a treadmill as a training modality, which is an activity typical for activities of daily living; thus, providing greater validity to our final results.

### Objectives

The main objective of this randomized controlled trial is to determine the effect of a high-intensity interval training program in comparison with a moderate-intensity continuous training program on VO_2max_. A secondary objective is to examine the effects of both training programs on systolic and diastolic blood pressure.

### Hypothesis

The primary hypothesis of this study is that compared with an 8 week moderate-intensity continuous training program, a high-intensity interval training program will have a significant effect on VO_2max_ in a group of 18–44-year-old men. A secondary hypothesis proposes that, in comparison with a moderate-intensity continuous training program, there is a significant positive effect on systolic and diastolic blood pressure with a high-intensity interval training program.

## Methods

### Study design

This study is a randomized controlled trial with parallel arms conducted and reported in accordance with CONSORT guidelines for non-pharmacologic treatment [31].

### Recruiting

Volunteers will be recruited through posters sent via email to the academic community throughout the Universidad de Antioquia, and located in various places on the university campus and nearby educational institutions, as well as via Facebook. Recruiting will finish once the number of volunteers required is complete. There will be no paid incentives to participate in this study.

### Research site

Assessments will be performed at the Laboratory for Physical Activity and Sports Sciences, Universidad de Antioquia. The interventions will be conducted at the sporting facilities of the Universidad de Antioquia.

### Eligibility criteria

Forty-four healthy but sedentary (≤150 min of aerobic exercise a week (as reported using the Global Physical Activity Questionnaire [32]) young men between 18 and 44 years old, who are enrolled in the General Social Security Health System in Colombia [33] will be recruited for this study. Prior to starting the program, all participants must accept their voluntary participation by signing an informed consent form. The Research Ethics Committee at the Universidad de Antioquia School of Public Health in Medellín, Colombia approved the study protocol. Subjects will be excluded if they are currently enrolled in a high-intensity interval training program, smoked, have a history of cardiovascular disease, coronary disease, arrhythmias, cardiac insufficiency, hypertension (history of hypertension, currently taking pharmacological agents, or blood pressure ≥ 140/90 mmHg), diabetes mellitus, are taking anticoagulants, beta blockers, calcium antagonists, bronchodilators, or steroids, or have cognitive impairments or neuromuscular or musculoskeletal disorders that could affect their participation in the exercise program. A physician specializing in sports medicine will evaluate all participants, and will confirm their participation in accordance with the study criteria.

### Randomization

The random allocation sequence will be created in permuted blocks of four and six using random allocation software [34]. Allocation concealment will be achieved using sequentially numbered, opaque sealed envelopes [35]. Volunteers will be assigned to an intervention or control group in a 1:1 ratio, depending on the order they enroll into the study (after evaluation of baseline outcomes). An independent researcher will conduct the process. Researchers in charge of recruiting, outcome evaluation, and analysis will be blinded in group assignment, and personnel in charge of evaluating and conducting interventions will be trained following the protocols designed for those objectives.

### Adherence strategies

After obtaining volunteers’ consent, volunteers will be sent reminders to attend their sessions through text messages, emails, and phone calls. Volunteers may contact the intervention coordinator and researchers 24 hours a day via email or by calling their cell phones.

### Information management

Files with participant’s information will be coded with individual identification codes. Data will be stored in a locked filing cabinet and in a password-protected computer, to which only researchers will have access. The computer password will be changed once a month throughout the duration of the research study; furthermore, a backup copy of all the information will be made once a month.

### Interventions

Twenty-four sessions of aerobic exercise on a treadmill, plus strength training exercises, will be completed as a non-differential co-intervention in both groups, based on the recommendations set forth by the American College of Sports Medicine for healthy beginners or intermediate-level adults [36]. Each participant will take part in three-weekly sessions of personalized training on alternate days at the same time of day for eight-weeks, supervised by a qualified trainer who is knowledgeable of the study protocol and procedures. Throughout each session, the intensity of the subject’s effort will be monitored using a Polar® FT1 heart rate monitor, and the compliance with each protocol will also be recorded, in forms designed for this purpose. Participants will warm up during the first 5 min on a treadmill at 50–60 % of maximal heart rate, and then follow the exercise protocol assigned to them (intervention or control), with a 3 min recovery period at 40–50 % of maximal heart rate. Finally, they will perform two sets of 8 to 12 repeats in a circuit training (non-differential co-intervention), consisting of eight strength training exercises mainly involving large muscle groups at 60–70 % of a one-repetition maximum, which equals a six or seven in the OMNI resistance exercise scale [37]. The objective of this portion of the intervention is simply to introduce these participants to the benefits of resistance training twice per week, as suggested by the American College of Sports Medicine [36]. We believe this portion of the intervention will not have an impact on any of the primary outcomes of our study.

The intervention group (high-intensity interval training), will undergo 15 bouts of 30 s loads at 90–95 % of maximal heart rate followed by a 60 s recovery at 50–55 % of baseline VO_2max_ with an effort:recovery ratio of 1:2. The intensity of each high-intensity interval training session will be reached and adjusted manually throughout the session by a trained investigator manipulating the speed of the treadmill to reach the target heart rate, while maintaining a constant 10 % elevation. It is worth highlighting that this intervention proposes short load times with double recovery time, which is different from those used in similar studies, which provided long load times with a recovery time similar to or less than the load time [11, 14, 24, 38, 39]. Other studies used an intervention with fewer but longer intervals [12, 40], and one study used a shorter load time [11]. Nevertheless, the total load time per session in those previous studies was greater than the time proposed in this randomized controlled trial, which is 21.5 min total (7.5 min at 90–95 % of maximal heart rate and 14 min of recovery). Furthermore, the intention of this (high-intensity interval training) intervention is to show whether a shorter total time of high-intensity loads per session can generate similar or higher increases in VO_2max_ than longer load interventions. This change may be important, considering the fact that current public health guidelines regarding the minimum dose of exercise required to improve cardiovascular health seem difficult to follow, owing to barriers related to users’ lack of time [28].

The control group (moderate-intensity continuous training) will do 40 min of continuous treadmill exercise at 65–75 % of maximal heart rate. Throughout the intervention, volunteers will be encouraged to continue with their regular daily routine and not engage in any other exercises different form the ones proposed in the research study. Furthermore, they will asked to wear an Omron® HJ-112 pedometer [41] to monitor their ambulatory daily activity.

### Evaluation of outcomes

The evaluation of primary and secondary outcome measures will follow the order established by the American College of Sports Medicine for health-related physical assessment [42] and will be conducted in the following order: blood pressure (systolic and diastolic) measurements, waist circumference, height, weight, body composition, and cardiovascular endurance test. The final evaluations will be conducted within 72 hours of the last training session. Prior to each evaluation, all participants will be asked to avoid alcohol and drink plenty of water 24 hours before the testing session. In addition, they will be asked to avoid caffeine 6 hours prior to the evaluation and to avoid any vigorous exercise. Participants will be encouraged to arrive well rested, having had 6–8 hours of sleep the night before, and to wear comfortable athletic clothing.

The evaluation of blood pressure will be conducted first during each testing session and will be performed using an Omron® M3 HEM-7200-E (Omron Healthcare, Co., Ltd., Kyoto, Japan) automatic blood pressure monitor [43] to measure both systolic and diastolic blood pressure measures. Participants will be asked to sit and remain seated for 5 min with their feet on the ground and their arms resting at the heart level [44]. Two blood pressure measurements will be performed on the left arm with a 1 min pause between measurements. The average of both values will be used as the final systolic and diastolic measurements.

To assess body composition, an Omron® HBF-510 (Omron Healthcare, Inc. Bannockburn, IL, USA) bioelectrical impedance machine will be used, following manufacturer protocols. Briefly, individuals will be instructed to wear light clothing, and stand barefoot on the device, which includes four electrodes on the feet and two electrodes on the handle. Participants will each be asked to hold the handle in their hands during the measurement, with the arms extended in front of them at eye level. The Omron® HBF-510 will measure weight, percentage of body fat, percentage of lean tissue and body mass index. To measure height, volunteers will be asked to stand on a stadiometer without shoes and with their heels together, while looking straight ahead. Participants will each be asked to inhale deeply, and hold their breath briefly while the evaluator slides the stadiometer set square to the top of their head and records the height in centimeters. Waist circumference will be measured following standard procedures, at the mid-point between the lower costal edge and the iliac crest [45].

Measurements of VO_2max_ will be obtained using a graded exercise test on a treadmill (Trackmaster® model TMX 425C) using a portable K4b^2^ gas analyzer (Cosmed Inc., IL, USA), which has been validated and utilized in previous studies [46–50]. The equipment will be warmed up and calibrated following standard procedures prior to each evaluation. Before every assessment and after each test, components will be cleaned and disinfected following the manufacturer’s recommendations, to guarantee valid measurements and to ensure participants’ safety.

To conduct the stress test, participants will warm up for 3 min at 5 km/h. Immediately following the warm-up period, the test will begin at a speed of 4 km/h and will increase by 1 km/h every minute with a constant 10 % gradient (5.7°) until maximal effort is achieved. To determine VO_2max_, the following criteria will be followed: respiratory quotient >1.10, maximal heart rate ≥95 %, following the 220-age equation [44], and reach a VO_2_ plateau despite an increase in load (VO_2_ does not increase or go less than 2 ml/(kg min)) [38, 51]. The test will end when the criterion is achieved or when the participant asks to stop the test because of fatigue or some other reason.

### Statistical considerations

#### Power and sample size

To calculate sample size, a 3.5 ml/(kg min) difference of measurements in VO_2max_ was considered as a minimum effect to decrease the risk of cardiovascular disease [4, 5] with standard deviations for the intervention group and the control group of 2.6 and 4.6, respectively [14], assuming a 95 % confidence level, 5 % alpha error, and 20 % beta error, with a 1:1 ratio between groups. Using Epidat software (version 4.0), it was found that a sample of 20 individuals per group would be needed, plus an estimated 10 % for possible losses.

#### Analysis plan

All analyses will be conducted according to the intention-to-treat principle, using a normality test, homoscedasticity test, or linearity test as necessary, to verify assumptions for the use of parametric statistics. As long as all assumptions of normality are met, analysis of covariance (ANCOVA) [52] will be used to control for the baseline values and adjust for possible confounding variables. In this regard, means and standard deviations will be used as summary measures. In the event that the requirements for parametric analysis are not met, a Mann–Whitney U test will be adopted, and values will be reported in medians and interquartile ranges. A two-tailed significance test with a *P* < 0.05 and a 95 % confidence level will be used. To manage missing data, multiple imputation procedures will be used to replace missing values for the primarily variables of interest, following the guidelines set forth by Stern *et al.* [53]. Briefly, multiple imputation procedures create several sets of plausible imputed data, based on the Bayesian approach, and combine the results obtained with each one to create a value for each missing data point. Stata software (version 13) will be used to carry out all statistical procedures.

## Discussion

VO_2max_ is considered one of the most important predictors of CVD mortality; in addition to smoking, high blood pressure (hypertension) and cholesterol disorders, low CRF levels increase the risk of CVD, is associated with morbidity and mortality from the same cause, as well as with all-cause mortality [4–6, 54–58]. Epidemiological evidence shows the link between VO_2max_ and its association with chronic conditions that trigger CVD, and subsequently increase the likelihood of death [56]. Similarly, high blood pressure is associated with increased risk of CVD [21]. Therefore, finding ways to increase VO_2max_ and reduce SBP and DBP it is of vital importance to public health. Recently the cardiorespiratory improvements obtained with HIIT have been examined; however, the optimal dose necessary requires further inquiry [20]; thus, it is important to develop RCT's as the one proposed in this study, which will clarify and provide insight to the cause-effect relationship between HIIT and VO_2max_, as well as HIIT and SBP-DBP. This research is novel since it is the first to examine a HIIT workout with a heavy load with a total length 7.5 minutes per session, compared with other studies using longer load periods [11, 13, 14, 38, 39]. This project is of utmost importance, as exercise adherence is typically hindered by the lack needed to complete a typically traditionally design exercise program [28]. Another strength of this study is the design used (RCT), which has the power to detect differences in the effect on VO_2max_ produced by a HIIT vs MICT program.

## Trial status

The call for participants started on April 25, 2015. We have enrolled 29 volunteers as of the submission of this manuscript. Of those 29, 18 have finished the interventions, and two withdrew from the study for personal reasons. The remaining volunteers are active. Recruitment of all volunteers is expected to finish in June 2016.

## Abbreviations

ANCOVA, analysis of covariance; CONSORT, Consolidated Standards of Reporting Trials; VO_2max_, maximal oxygen uptake.
